# Randomized study of the safety, pharmacokinetics, and bronchodilatory efficacy of a proprietary glycopyrronium metered-dose inhaler in study patients with chronic obstructive pulmonary disease

**DOI:** 10.1186/1471-2466-14-118

**Published:** 2014-07-16

**Authors:** Stephen Rennard, Charles Fogarty, Colin Reisner, Carlos Fernandez, Tracy Fischer, Michael Golden, Earl St Rose, Patrick Darken, Gregory Tardie, Chadwick Orevillo

**Affiliations:** 1 University of Nebraska Medical Center, Physicians Internal Medicine at the Durham Outsubject Center, Omaha, NE, USA; 2 Spartanburg Medical Research, Spartanburg, SC, USA; 3 Pearl Therapeutics, Inc., Morristown, NJ, USA

**Keywords:** Chronic obstructive pulmonary disease, Glycopyrronium, Porous particle technology, Long-acting anticholinergic, Long-acting muscarinic antagonist

## Abstract

**Background:**

Bronchodilator medications are central to the symptomatic management of chronic obstructive pulmonary disease (COPD). Metered-dose inhalers (MDIs) are the most commonly used devices to deliver treatment to patients with COPD and asthma, comprising approximately 70% of bronchodilator prescriptions. Proprietary porous-particle technology permits the formulation of long-acting muscarinic antagonists, long-acting β_2_-agonists, and a combination of both in hydrofluoroalkane (HFA) MDIs, providing a solution to formulation challenges inherent to the development of HFA MDIs, which have contributed to the development of dry-powder inhalers.

**Methods:**

In this randomized, double-blind, 4-period, 6-treatment, placebo- and active-controlled, multicenter, crossover study, 4 ascending single doses of a proprietary glycopyrronium (GP) MDI were evaluated compared with Placebo MDI and open-label tiotropium (TIO) in study patients with COPD. Thirty-three study patients were enrolled and received single-dose administration of 4 of the 6 treatments (Placebo MDI, TIO 18 μg, or GP MDI at 14.4, 28.8, 57.6, and 115.2 μg ex-actuator) with an interval of 1 to 3 weeks between doses. The primary efficacy endpoint was peak change in forced expiratory volume in 1 second (FEV_1_).

**Results:**

All 4 doses of GP MDI showed statistically superior efficacy compared with Placebo MDI for peak FEV_1_ (differences of 146 to 248 mL; *P* < .001), with a clear dose ordering of the response. Statistically significant differences compared with Placebo MDI were noted at almost all doses for the secondary FEV_1_ parameters (*P* ≤ .049) except 24-hour trough FEV_1_ at 28.8 μg. All doses were safe and well tolerated in this study; the most frequently reported adverse event was dry mouth (0–14.3% across doses; 9.5% for Placebo MDI, and 9.1% for TIO).

**Conclusions:**

This study demonstrated superior bronchodilatory efficacy of GP MDI compared with Placebo MDI at all doses tested, and no serious adverse events were reported. This study supports the further evaluation of GP MDI in study patients with COPD. In addition, these findings indicate that the correct dosage of glycopyrronium is no more than 115.2 μg total daily dose, or 57.6 μg twice daily based on comparisons with the active comparator.

**Trial registration:**

This clinical trial was registered on ClinicalTrials.gov, Identifier: NCT00871182.

## Background

Bronchodilator medications are central to the symptomatic management of chronic obstructive pulmonary disease (COPD) [1]. Inhaled short- and long-acting muscarinic antagonists (SAMAs and LAMAs), such as ipratropium and tiotropium bromide, respectively, are among the main classes of bronchodilators used for the treatment of COPD and have been shown to improve lung function and reduce COPD symptoms [2]. Metered-dose inhalers (MDIs) are the most commonly used devices to deliver treatment to patients with COPD and asthma, comprising approximately 70% of bronchodilator prescriptions [2,3]. Although they are easy to use and are well-accepted, efforts to comply with federal laws mandating the phasing-out of chlorofluorocarbon propellants have encountered technical barriers in the creation of a stable formulation, which have led several companies to instead pursue the commercialization of dry-powder inhalers (DPIs).

Hollow, porous-particles added to HFA MDIs provide a mechanism for stabilizing suspensions of inhaled drugs, leading to improved physical stability, ability to formulate very low doses, consistent dose-to-dose performance, and high fine-particle fraction (FPF) [4,5]. The large area of the hollow, porous particles allows therapeutic agents to be adsorbed and delivered into the lungs during inhalation. The particles themselves are comprised of distearoyl-phosphatidylcholine (DSPC), a naturally occurring lung surfactant that is used frequently in approved pharmaceutical preparations, and CaCl_2_. The initial exploration of these respirable porous particles as a delivery platform for inhaled bronchodilator therapy was performed with formoterol as a monotherapy. Another advantage offered by the porous-particle technology includes relatively uniform aerodynamic particle size distribution allowing delivery of drug to the lower respiratory tract [4-7] with minimized oropharyngeal exposure.

Glycopyrronium (GP) is an antimuscarinic drug that has been approved by the U.S. Food and Drug Administration for systemic administration in multiple clinical scenarios, including as a preoperative agent to reduce the volume of saliva and acidity of gastric secretions [8,9]. It is a quaternary ammonium derivative that when inhaled results in minimal mucosal absorption and systemic side effects [10,11]. A dry-powder inhalation formulation of glycopyrronium recently approved in Europe and Japan has demonstrated significant and sustained bronchodilator efficacy over a 24-hour period as well as a positive safety and tolerability profile [12,13].

The present study represents the first clinical study of a glycopyrronium metered-dose inhaler (GP MDI) developed using a proprietary lipid-based porous-particle. This study was designed to evaluate the efficacy and safety of 4 doses of GP MDI to serve as the basis for selecting the dose(s) that produce a consistent 12- and/or 24-hour duration of action for more definitive studies. Tiotropium bromide 18 μg [(Spiriva® delivered via the Handihaler®; Boehringer Ingelheim Pharmaceuticals, Ridgefield, Connecticut)(TIO)] [14] was included as an open-label active comparator.

## Methods

This was a randomized, double-blind, 4-period, 6-treatment, placebo- and active-controlled, incomplete block, crossover, multicenter study to evaluate the single administration of 4 ascending single doses of GP MDI compared with Placebo MDI and TIO as an active control in study patients with COPD, and was conducted between March, 2009 and September, 2009. The particles administered by this device have a mean aerodynamic diameter of 3.5 μm (GP MDI 14.4 μg in monotherapy) [5]. It should be noted that Pearl Therapeutics has recently revised the nomenclature for GP MDI to refer to the active moiety, glycopyrronium, rather than the bromide salt form previously referred to as glycopyrronium bromide (also known as glycopyrrolate). The doses tested in the current study are equivalent to 115.2, 57.6, 28.8 and 14.4 μg of glycopyrronium. This change does not reflect a change to the formulation of GP MDI, only in the expression of the strength/dose. To aid in the selection of doses for further development, a marketed open-label active comparator was included in this study (TIO).

The conduct of this study (PT0010801) complies with the Declaration of Helsinki, and approvals were obtained through an accredited Institutional Review Board (Independent IRB, Plantation, FL, USA). Written informed consent was obtained from each study patient prior to entry into the trial. The study was listed on all appropriate clinical trial registries, including the United States (US) National Institutes of Health’s ClinicalTrials.gov (NCT00871182).

### Study patients

Eligible patients were 40 to 75 years of age with an established clinical history of COPD; current or prior history of at least 10 pack-years of cigarette smoking; measured post-ipratropium forced expiratory volume in 1 second (FEV_1_)/forced vital capacity (FVC) ratio of ≤0.70; measured post-ipratropium FEV_1_ ≥ 50% and ≤85% of predicted normal values; and demonstrated reversibility to ipratropium (>200 mL improvement and/or >12% and >150 mL improvement from baseline FEV_1_ 30 minutes following administration of 4 puffs of ipratropium).

Key exclusion criteria were a primary diagnosis of asthma, history of significant diseases other than COPD, acute worsening of COPD that required treatment within 6 weeks prior to screening or between the screening and baseline visits, symptomatic prostatic hypertrophy or bladder neck obstruction, known narrow-angle glaucoma, uncontrolled hypertension, or clinically significant electrocardiogram (ECG) abnormalities.

Study patients meeting the entry criteria who were taking certain COPD medications at screening (TIO, oral β_2_-agonists, long-acting β_2_-agonists [LABAs], combination corticosteroid/LABA products, theophylline, leukotriene inhibitors [zafirlukast, montelukast, zileuton], or cromoglycate and/or nedocromil) discontinued these medications for the duration of the trial and were switched to short-acting bronchodilators (ipratropium, albuterol, or ipratropium/albuterol combination), with or without an inhaled corticosteroid. During the study, 3.1% (n = 1) of study subjects were using concomitant inhaled corticosteroids. Patients that were not taking any of the COPD medications, and were previously maintained on albuterol, ipratropium or a combination thereof, with or without an inhaled corticosteroid (ICS) for at least one week prior to screening (Visit 1a), were permitted to proceed directly to Visit 2, providing they met all entry criteria. Study patients previously treated with a maintenance dose of an ICS that was not administered as a fixed dose combination together with a LABA were permitted to continue the ICS, providing the daily dose did not exceed 1,000 μg of fluticasone or equivalent, and they had been maintained on a stable dose for at least 2 weeks.

To ensure that study patients were stable on the revised treatment regimen, they were required to return for a second screening visit at least 1 week but not longer than 3 weeks after altering maintenance medication. Study patients were required to withhold all COPD medications (including inhaled corticosteroids) for at least 6 hours before the baseline visit and all subsequent study treatment visits.

At Visit 2, study patients were randomized to 1 of 6 possible treatment sequences. Each sequence included single administration of 4 of the 6 treatments evaluated in the study: GP MDI at 14.4, 28.8, 57.6, and 115.2 μg ex-actuator, Placebo MDI, and TIO 18 μg (active comparator). Each sequence included 2 or 3 GP MDI doses, administered in ascending order with a randomized position of TIO and/or Placebo MDI in the sequence. Patients returned to the clinic for treatment at Visits 3, 4, and 5 and for the final visit (Visit 6; approximately 1 week after Visit 5). There was a washout interval between doses of at least 1 week and no more than 3 weeks.

### Study assessments and variables

On each of the 4 study drug dosing visits, spirometry was conducted 60 and 30 minutes prior to study drug administration; the average of these 2 assessments was used to establish test-day baseline FEV_1_. Spirometry was obtained at 15 and 30 minutes, and 1, 2, 4, 6, 8, 10, 12, 16, 22, 23, and 24 hours post-dosing. The primary efficacy endpoint was peak improvement in FEV_1_ relative to test-day baseline, which was compared for each dose of GP MDI relative to Placebo MDI. The secondary efficacy endpoints included FEV_1_ area under the curve (AUC) for the 12, 24, and 12- to 24-hour periods post-dosing relative to test-day baseline: FEV_1_ AUC_0-12_, FEV_1_ AUC_0–24_, and FEV_1_ AUC_12–24_, trough FEV_1_ (tFEV_1_), and the time to onset of action (≥10% improvement in mean FEV_1_ from test-day baseline). Trough FEV_1_ at 24 hours is defined as the mean of the FEV_1_ assessments taken at 23 and 24 hours post-study drug administration. AUC values were normalized in order to provide results in liter (L) units by dividing by the length of time included in each interval. Pharmacokinetic (PK) sampling was performed at each treatment visit and was conducted at specified time points up to 24 hours post-dose. Safety analyses included adverse events (AEs) and serious adverse events (SAEs), hematology and chemistry laboratory assessments, ECGs, vital signs, and monitoring for paradoxical bronchospasm and symptoms of dry mouth.

### Statistical analyses

Published data suggest that it is reasonable to expect a difference between investigational doses and placebo in peak FEV_1_ that will exceed 0.15 L. Thus, if the within-patient standard deviation does not exceed 0.20 L there will be at least 80% power with N = 16 for the pairwise comparisons [15]. The primary efficacy analyses were the estimation of the pairwise differences along with 95% two-sided confidence intervals (CIs) for each GP MDI dose vs Placebo MDI. Contrasts were estimated using mixed-model analysis of variance for repeated measures (MMRM) with subject as a random effect. A Wilcoxon rank sum test was used to assess differences in time-to-onset of effect.

Multiplicity was controlled for the primary endpoint by a sequential procedure where GP MDI 115.2 μg was first compared with Placebo MDI using a two-sided alpha level of 5%. If this was significant, then GP MDI 57.6 μg was compared with Placebo MDI using the same alpha level. This sequential testing approach was continued for comparisons of lower doses with Placebo MDI in descending order. In addition, there was a linear test of trend for the dose response that was prespecified to be interpreted inferentially only if GP MDI 115.2 μg was significant compared with Placebo MDI. There were no further adjustments for multiple comparisons and nominal significance is reported for all other comparisons based on two-sided *P* values < .05.

PK parameters were derived from the glycopyrronium concentration-time data. Plasma PK parameters of glycopyrronium were calculated using non-compartmental models. Concentrations below the limit of quantitation were set to zero. Descriptive statistics were reported for each PK parameter.

## Results

### Disposition and baseline characteristics

**Figure 1:**
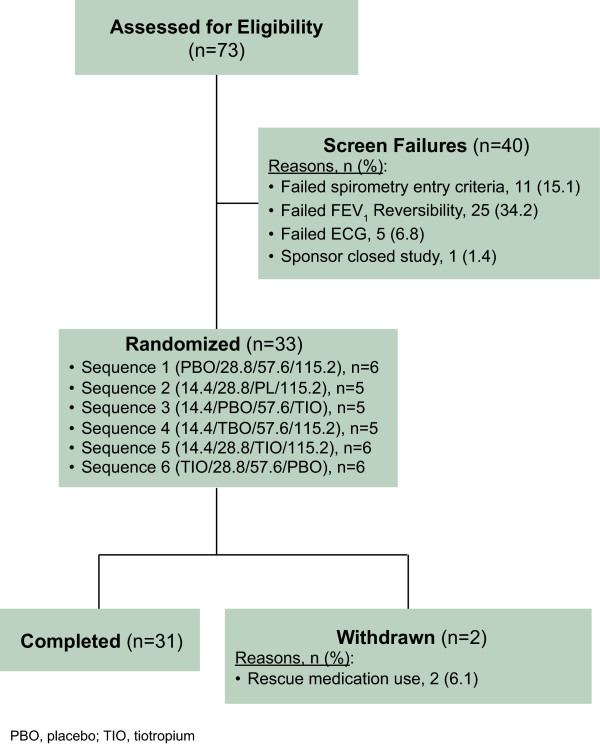
**Study patient disposition; reports the overall enrollment, allocation, and follow-up for study patients.** Two patients were excluded for both failed FEV_1_ reversibility and a failed ECG, making the total number of patients who failed screening 40.

A total of 73 subjects were screened for the study. Of these 25 (34.2%) were not eligible because of the reversibility criteria. Of those eligible, 33 study patients were enrolled in this study (Figure 1). No patients meeting the entry criteria were taking leukotriene inhibitors (zafirlukast, montelukast, zileuton) or cromoglycate and nedocromil. Two study patients were withdrawn due to rescue medication use during a test day. The majority of study patients were male (58%) and Caucasian (97%). Other demographic and baseline clinical characteristics are shown in Table 1. The intent-to-treat/safety population (N = 33) included all study patients who were randomized and received at least one dose of study treatment and was used for demographic and safety analyses. A modified intent-to-treat (mITT) population (n = 30) that excluded 3 randomized study patients due to protocol violations (rescue medication use) was used for PK and efficacy analyses.

**Table 1:** Demographic and baseline characteristics (safety population)

**Characteristics**	**N = 33**
Race, n (%)	
Caucasian	32 (97%)
Hispanic or Latino	1 (3%)
Gender, n (%)	
Male	19 (58%)
Age, years	
Mean (SD)	59.0 (6.7)
Median (Range)	58.1 (44–71)
Smoking Status, n (%)	
Current Smoker	18 (55%)
Years Ago Quit, n	15
Mean (SD)	10.2 (7.7)
Median (Range)	9.5 (0–27)
Number of Years Smoked	
Mean (SD)	38.6 (10.5)
Median (Range)	40.0 (13–57)
FEV_1_, L/sec, prebronchodilator	
Mean (SD)	1.6 (0.5)
Median (Range)	1.5 (0.9–3.0)
FEV_1_, % predicted, prebronchodilator	
Mean (SD)	50.5 (9.9)
Median (Range)	47.3 (35.5–69.3)
FEV_1_, % predicted, postbronchodilator	
Mean (SD)	60.6 (10.3)
Median (Range)	57.6 (43.0–80.5)

FEV_1_ = forced expiratory volume in 1 second; SD = standard deviation; L=liter

### Efficacy

#### Primary efficacy variable

**Figure 2:**
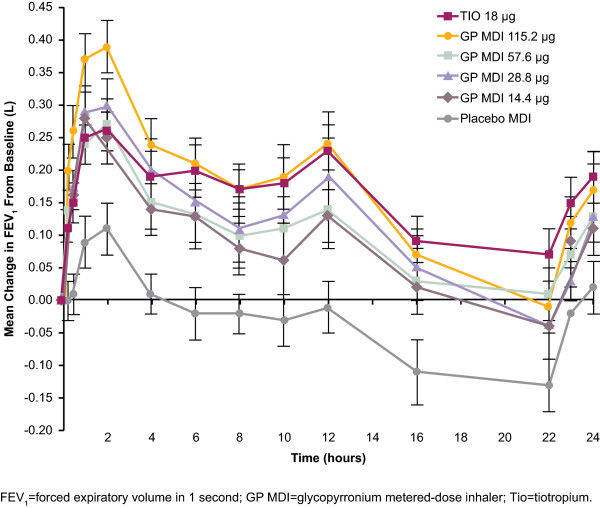
**Mean (±standard error) change from baseline in FEV**_**1 **_**over 24 hours by treatment; represents the results for the peak change in FEV**_**1 **_**(the primary efficacy endpoint) as well as the change in FEV**_**1 **_**from test-day baseline over time.**

The mean change in FEV_1_ from test-day baseline over time is illustrated in Figure 2. All 4 doses of GP MDI demonstrated statistically superior efficacy compared with Placebo MDI (*P* < 0.001) for peak change in FEV_1_ (Table 2). There was a clear dose ordering of the peak FEV_1_ response, with the 115.2 μg dose presenting the greatest difference from placebo (248 mL). TIO 18 μg also demonstrated statistically superior efficacy compared with Placebo MDI (P < 0.001) for peak change in FEV_1_.

**Table 2:** **Mean peak change from baseline FEV**_**1**_**(L) by treatment compared with placebo (mITT population)**

	**PBO MDI**	**GP MDI 14.4 μg**	**GP MDI 28.8 μg**	**GP MDI 57.6 μg**	**GP MDI 115.2 μg**	**TIO 18 μg**
Peak Change	0.182	0.328	0.340	0.362	0.430	0.380
Comparison vs PBO						
Contrast Difference^a^	N/A	0.146	0.158	0.180	0.248	0.198
SE	N/A	0.040	0.039	0.039	0.040	0.040
90% CI	N/A	0.066, 0.225	0.081, 0.235	0.103, 0.257	0.168, 0.327	0.119, 0.278
*P*^b^	N/A	<0.001	<0.001	<0.001	<0.001	<0.001

^a^Contrast difference [Treatment 1-Treatment 2]; ^b^*P* from mixed-model analysis of variance.

CI = confidence interval; FEV_1_ = forced expiratory volume in 1 second; GP MDI = glycopyrronium metered-dose inhaler; L = liters; mITT = modified intent-to-treat; N/A = not applicable; PBO MDI = placebo metered-dose inhaler; SE = standard error; TIO = tiotropium.

#### Secondary efficacy variables

**Figure 3:**
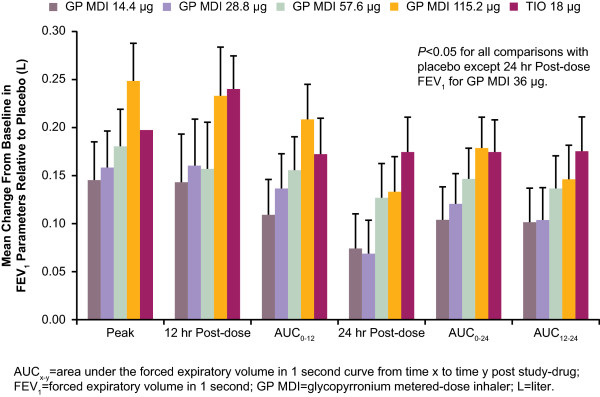
**Adjusted Mean (±standard error) change from baseline in FEV**_**1**_**parameters relative to placebo; provides a concise depiction of the mean change from baseline in peak FEV**_**1**_**, 12-hour FEV**_**1**_**, FEV**_**1**_**AUC**_**0–12**_**, 24 hour FEV**_**1**_**, FEV**_**1**_**AUC**_**0–24**_**, FEV**_**1**_**AUC**_**12–24**_**.**

Each of the GP MDI doses exhibited a statistically significantly greater (*P* ≤ 0.049) mean change from test-day baseline compared with Placebo MDI for 12- and 24-hour tFEV_1_, FEV_1_ AUC_0-12_, FEV_1_ AUC_0–24_, and FEV_1_ AUC_12–24_, with the single exception of the GP MDI 28.8 μg comparison for the 24-hour tFEV_1_ (*P* = 0.059) (Figure 3). GP MDI demonstrated a more rapid onset of action compared with TIO 18 μg, with median time to ≥10% improvement in FEV_1_ of 0.5 hours or less for all doses of GP MDI evaluated, compared with approximately 1 hour for TIO 18 μg.

### PK

**Figure 4:**
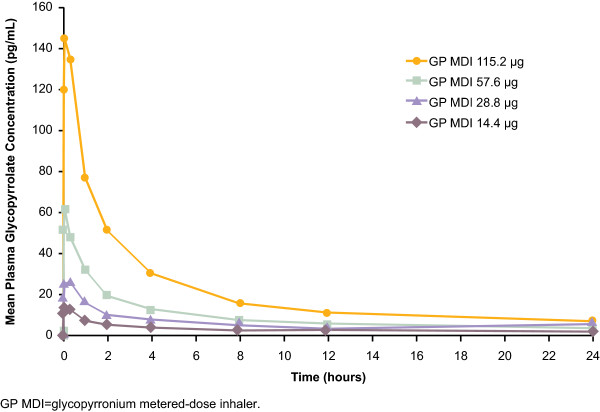
Mean plasma glycopyrronium concentrations over time.

Mean plasma glycopyrronium concentrations over time are presented in Figure 4. Overall, exposure (both maximum plasma concentration [C_max_] and AUC) increased in a proportional manner with dose (Table 3). C_max_ was reached rapidly (usually within 6 minutes of dosing). Apparent oral clearance and volume of distribution appeared relatively consistent across doses.

**Table 3:** Summary of glycopyrronium pharmacokinetic parameters (mITT population)

**Parameter**	**Arithmetic mean (CV%)**			
	**GP MDI 14.4 μg**	**GP MDI 28.4 μg**	**GP MDI 57.6 μg**	**GP MDI 115.2 μg**
**N**	18	18	20	20
AUC_0–24_ (pg•h/mL)	34.5 (101.2)	120 (67.2)	202 (74.5)	498 (82.0)
AUC_0–12_ (pg•h/mL)	31.9 (88.8)	89.9 (57.7)	163 (70.6)	398 (79.8)
AUC_12–24_ (pg•h/mL)	4.34 (250.5)^b^	32.0 (147.0)^d^	41.0 (91.3)^e^	102 (100.9)^f^
AUC_0-tlast_ (pg•h/mL)	30.7 (112.5)	113 (74.4)	196 (78.8)	491 (84.4)
AUC_0-inf_ (pg•h/mL)	66.2 (72.2)^c^	127 (68.7)^b^	252 (70.8)^e^	598 (84.0)^f^
C_max_ (pg/mL)	15.6 (72.0)	27.3 (51.5)	62.9 (72.3)	160 (73.8)
t_max_^a^ (h)	0.333 (0.0330, 0.350)	0.100 (0.0330, 0.383)	0.100 (0.0330, 0.917)	0.100 (0.0330, 0.933)
t_1/2_ (h)	5.09 (82.0)^c^	6.28 (62.6)^b^	8.76 (59.4)^e^	9.61 (36.6)^f^
CL/F (L/h)	416 (77.0)^c^	494 (89.6)^b^	510 (81.3)^e^	422 (75.6)^f^
Vz/F (L)	1995 (32.2)^c^	3320 (56.9)^b^	4627 (50.9)^e^	4697 (49.7)^f^

^a^Median (Min, Max); ^b^n = 11; ^c^n = 10; ^d^n = 12; ^e^n = 19; ^f^n = 16.

AUC_x-y_ = area under the concentration-time curve from time x to time y; CL/F = apparent oral clearance; C_max_ = maximum plasma concentration; CV% = coefficient of variation; GP MDI = glycopyrronium metered-dose inhaler; t_1/2_ = apparent terminal elimination half-life; t_max_ = time to maximum concentration; Vz/F = apparent volume of distribution; mITT=modified intention to treat.

### Safety

The most frequently reported AE was dry mouth (Table 4). All other AEs were reported only once or twice during the study and were distributed across the treatments. Dry mouth was also the most frequently reported drug-related AE. The only other AEs besides dry mouth reported as being drug-related were dyspnea (GP MDI 28.8 μg, 1 study patient) and paradoxical bronchospasm, defined as a reduction in FEV_1_ of >20% from test-day baseline with associated symptoms of wheezing, shortness of breath or cough (GP MDI 115.2 μg, 1 patient; mild in severity on the day following treatment). No patient experienced an SAE during the study, and no study patient discontinued due to an AE. Changes in hematology, clinical chemistry, vital signs, physical examination findings, and ECGs were small, and no treatment-related trends were observed.

**Table 4:** Number (%) of study patients reporting AEs (safety population)

**Preferred term**	**PBO MDI**	**GP MDI**	**GP MDI**	**GP MDI**	**GP MDI**	**TIO**
	**n = 21**	**14.4 μg**	**28.8 μg**	**57.6 μg**	**115.2 μg**	**18 μg**
	**n (%)**	**n = 21**	**n = 23**	**n = 21**	**n = 21**	**n = 22**
		**n (%)**	**n (%)**	**n (%)**	**n (%)**	**n (%)**
**One or more AEs**	4 (19.0)	3 (14.3)	3 (13.0)	5 (23.8)	3 (14.3)	4 (18.2)
Dry mouth	2 (9.5)	1 (4.8)	0	3 (14.3)	1 (4.8)	2 (9.1)
Oropharyngeal pain	0	1 (4.8)	0	0	1 (4.8)	0
Bronchospasm paradoxical	0	0	0	0	1 (4.8)	0
Nasopharyngitis	0	0	0	1 (4.8)	0	0
Urinary tract infection	0	0	0	1 (4.8)	0	0
Vessel puncture site hematoma	0	0	1 (4.3)	0	0	1 (4.5)
Sinusitis	0	0	1 (4.3)	0	0	0
Headache	0	0	1 (4.3)	0	0	0
Insomnia	0	0	1 (4.3)	0	0	0
Dyspnea	0	0	1 (4.3)	0	0	0
Hypertension	0	0	1 (4.3)	0	0	0
Dizziness	0	1 (4.8)	0	0	0	0
Cough	0	1 (4.8)	0	0	0	0
Diarrhea	0	0	0	0	0	1 (4.5)
Umbilical hernia	1 (4.8)	0	0	0	0	0
Gastroenteritis	1 (4.8)	0	0	0	0	0
Gastrointestinal viral infection	1 (4.8)	0	0	0	0	0

AE = adverse event; DPI = dry powder inhaler GP MDI=glycopyrronium metered-dose inhaler; PBO = placebo; TIO = tiotropium.

## Discussion

The current study assessed GP administered by an MDI using a proprietary lipid-based porous-particle. The FEV_1_ data obtained during this study indicate that a single-dose of GP MDI yields statistically significant, dose-dependent, and clinically relevant bronchodilation at all doses compared with Placebo MDI. In terms of peak improvement in FEV_1,_ the efficacy of GP MDI 57.6 and 115.2 μg bracketed that of TIO 18 μg. Although both GP MDI 14.4 and 28.8 μg demonstrated clinically relevant and statistically significant peak improvement in FEV_1_ compared with Placebo MDI, the magnitude of these improvements was numerically lower than those observed with TIO 18 μg.

The primary efficacy results are corroborated by the secondary efficacy variables: 12- and 24-hour post-dose FEV_1_, FEV_1_ AUC_0-12_, FEV_1_ AUC_0–24_, and FEV_1_ AUC_12–24_, with statistically significant greater mean changes from test-day baseline compared with Placebo MDI for all comparisons, with the exception of GP MDI 28.8 μg for 24-hour post-dose FEV_1_. Point-estimates for the majority of FEV_1_ parameters for GP MDI 57.6 and 115.2 μg were within ± 50 mL compared with TIO. All GP MDI doses demonstrated a rapid onset of action, and the 115.2 μg dose demonstrated a significantly faster onset than TIO 18 μg. With regard to the duration of action of GP MDI, several observations confirm that GP MDI is appropriate for twice daily (BID) dosing. For all GP MDI doses, the changes from baseline in FEV_1_ compared to Placebo MDI at 12 hours post-dose were appreciably greater than the changes at 24 hour post-dose, while these values were more consistent for TIO compared to Placebo MDI. Similarly, FEV_1_ AUC_0–12_ was greater than FEV_1_ AUC_12–24_ for all GP doses compared with Placebo MDI, while for TIO the difference from Placebo MDI in FEV_1_ AUC maintained close to 1:1 ratio across the 0- to 12- and 12- to 24-hour intervals. All doses of GP MDI were safe and well tolerated. The most frequently reported AE was dry mouth, which was reported for a similar proportion of study patients following Placebo MDI, GP MDI, and TIO (0–14.3% of patients).

Placing the data from the current study into context with those of existing glycopyrronium formulations is warranted. A 14-day PK study of NVA237 (Seebri® Breezehaler®, Novartis Europharm Limited, Horsham, West Sussex, United Kingdom) in doses of 25, 40, 100, and 200 μg found that the median time to reach maximal plasma concentration (t_max_) was similar between NVA237 (5–6.5 minutes post-inhalation) [16], to the 6 minutes for GP MDI 28.8 to 115.2 μg observed in the current study. The mean terminal elimination half-life (t_1/2_) of NVA237 is reported to range between 13 and 22 hours [16] compared with 6.3 to 9.6 hours for GP MDI 28.8 to 115.2 μg. It remains subject to debate whether these PK traits are generalizable to specific benefits in lung function which favor once-daily (QD) or BID dosing.

In comparing the efficacy of GP MDI observed in the current study with that of NVA 237 50 μg administered QD in the Glycopyrronium bromide in COPD airWays clinical Study 1 (GLOW1) trial; treatment with NVA237 resulted in an improvement in 24-hr post dose FEV_1_ (average of mean 23 hours 15 minutes, and 23 hours 45 minutes post-dose values) of 105 mL after initial dosing compared with placebo (*P* < .001) [17]. In the GLOW2 study [18], study patients received either NVA 237 50 μg, TIO 18 μg or placebo once-daily (QD), the differences from placebo in 24-hr post-dose FEV_1_ after initial dosing were 91 and 83 mL for NVA 237 and TIO, respectively. For the current study, GP MDI 115.2, 57.6, 28.8, and 14.4 μg resulted in an improvement in 24-hour post-dose FEV_1_ compared with placebo of 133, 127, 68, and 74 mL, respectively (*P* < .05). The 12-hour post-dose FEV_1_ for GP MDI 115.2, 57.6, 28.8, and 14.4 μg BID in the current study compared with placebo was 233, 157, 160, and 143 mL, respectively (all *P* < .001). It should be noted that in the current study, the screening post-bronchodilator percentage predicted FEV_1_ was 60.6% compared with 54% for GLOW1 and 56% for GLOW2. Such patients may be expected to demonstrate a greater post-bronchodilator response. Of further note, since subjects in the current study were required to be reversible, while most groups of COPD patients benefit clinically by typical LAMAs [1], subgroup data for QD umeclidinium suggest that subjects who are more reversible or who are current smokers may demonstrate somewhat greater FEV_1_ responses to treatment with LAMA [19].

In the GLOW2 study, for FEV_1_ AUC_0–12_ on Day 1, the difference from placebo for NVA237 was 159 mL, 32 mL above TIO [18], whereas in the current study the difference from Placebo MDI for GP MDI was 208, 155, 137, and 109 mL for 115.2, 57.6, 28.8, and 14.4 μg, respectively, in comparison to 172 mL for TIO. Also in GLOW2, the Day 1 improvement in peak FEV_1_ for NVA 237 compared to placebo was 200 mL, 47 mL greater than TIO. Whereas in the current study, the peak change from baseline in Peak FEV_1_ for GP MDI compared to Placebo MDI was 248, 180, 158, and 146 mL for 115.2, 57.6, 28.8, and 14.4 μg, respectively, in comparison with 198 mL for TIO.

Twice-daily dosing is of particular interest for a companion combination product containing glycopyrronium and the LABA formoterol fumarate, which is currently in development using the same porous-particle–based MDI technology. The use of a LAMA in combination with a LABA has been advocated in patients with COPD as airflow obstruction becomes more severe [20-22]. As a co-suspension platform, the porous-particle technology produces efficient and stable single, dual, and triple combination MDIs without a coformulation induced change in aerosol performance across product types.

### Study limitations

This study was conducted in COPD patients with demonstrated reversibility to a SAMA. Reversibility in a COPD population is variable. Many “non-reversible” patients will experience effects of smaller magnitude, but may still benefit; therefore, results should be confirmed in a broader COPD population. The study assessed the dose response following single dose administration across a wide range of GP MDI doses. For initial assessment of dose response, single dose administration is appropriate, however these findings should be confirmed.

Clinical benefits will require chronic-dosing that must be evaluated in appropriately designed longer-term studies. Finally, the relatively small sample size precludes many comparisons with the active comparator and provides limited information about associated rare events.

## Conclusions

This study was designed to evaluate the efficacy and safety of 4 doses of GP MDI so that the dose(s) that produce a consistent 12- and/or 24-hour duration of action can be selected for more definitive studies (phase 2b/phase 3). Glycopyrronium MDI 14.4, 28.8, 57.6, and 115.2 μg demonstrated statistically significant and clinically relevant superior bronchodilator efficacy compared with Placebo MDI in study patients with mild to moderate COPD. The overall profile of GP MDI based on 12-hr post-dose FEV_1_ compared with 24-hr post-dose tFEV_1_, and AUC_0–12_ compared with AUC_12–24_ support a BID dosing regimen at all doses evaluated. These same comparisons confirmed TIO as a QD drug. Based on the results of the current study, the candidate doses of GP MDI for further evaluation were total daily doses of 57.6 or 115.2 μg, administered as 28.8 or 57.6 μg given BID.

## Abbreviations

AE: Adverse event; AUC: Area under the curve; BID: Twice daily; CI: Confidence interval; C_max_: Maximum plasma concentration; COPD: Chronic obstructive pulmonary disease; DPI: Dry-powder inhaler; DSPC: Distearoyl-phosphatidylcholine; ECG: Electrocardiogram; FEV_1_: Forced expiratory volume in 1 second; FPF: Fine-particle fraction; FVC: Forced vital capacity; GP MDI: Glycopyrronium metered-dose inhaler; GP: Glycopyrronium; HFA: Hydrofluoroalkane; L: Liter; LABA: Long-acting beta-agonist; LAMA: Long-acting muscarinic antagonist; MDI: Metered-dose inhaler; mITT: Modified intent-to-treat; MMRM: Mixed- model analysis of variance for repeated measures; PBO MDI: placebo metered-dose inhaler; PD: Pharmacodynamic; PK: Pharmacokinetic; QD: Once daily; QID: Four times daily; SAE: Serious adverse event; SAMA: Short-acting muscarinic antagonist; t_1/2_: Elimination half-life; tFEV: Trough forced expiratory volume in 1 second; t_max_: Time to reach maximal plasma concentration.

## Competing interests

Dr Rennard is a consultant and investigator for Pearl Therapeutics, Inc.

Dr Fogarty is a consultant and investigator for Pearl Therapeutics, Inc.

Dr Reisner is an employee of Pearl Therapeutics, Inc.

Dr Fernandez is an employee of Pearl Therapeutics, Inc.

Dr Darken is an employee of Pearl Therapeutics, Inc.

Dr Fischer is an employee of Pearl Therapeutics, Inc.

Mr Golden is an employee of Pearl Therapeutics, Inc.

Mr St. Rose is an employee of Pearl Therapeutics, Inc.

Dr Tardie is an employee of Pearl Therapeutics, Inc.

Mr Orevillo is an employee of Pearl Therapeutics, Inc.

## Authors’ contributions

SR was a study investigator, and contributed to the development of the manuscript. CFo was a study investigator, and contributed to the development of the manuscript. CR oversaw the design of the study oversaw the conduct of the study, and contributed to the development of the manuscript. CFe contributed to the design of the study, oversaw the conduct of the study, and contributed to the development of the manuscript. TF contributed to the design of the study, oversaw the conduct of the study, and contributed to the development of the manuscript. MG contributed to the design, conduct and oversight of the study, and to the development of the manuscript. ESR oversaw the conduct of the study, and contributed to the development of the manuscript. PD contributed to the interpretation of results and development of the manuscript. GT contributed to the development and submission of the manuscript. CO contributed to the design of the study, oversaw the conduct of the study, and contributed to the development of the manuscript. All authors read and approved the final manuscript.

## Authors’ information

SR is a consultant and investigator of Pearl Therapeutics. CFo is a consultant and investigator of Pearl Therapeutics. CR is the executive vice president and chief medical officer of Pearl Therapeutics. CFe is a senior medical director of Pearl Therapeutics. TF is a senior director, regulatory affairs of Pearl Therapeutics. MG is the vice-president, regulatory affairs and quality assurance of Pearl Therapeutics. ESR is a Senior Director and head of clinical operations of Pearl Therapeutics. PD is the vice-president, biostatistics of Pearl Therapeutics. GT is a publications manager of Pearl Therapeutics. CO is a vice-president of clinical development and the head of medical communications of Pearl Therapeutics.

## Pre-publication history

The pre-publication history for this paper can be accessed here:

http://www.biomedcentral.com/1471-2466/14/118/prepub
